# Highly Selective Colorimetric and Smartphone-Based Paper Assay Using Malic Acid–Functionalized Silver Nanoparticles for Thiram Detection

**DOI:** 10.1021/acsomega.5c00575

**Published:** 2025-05-13

**Authors:** Kuan-Hsun Chen, Wei-Yu Wang, Cho-Chun Hu, Tai-Chia Chiu

**Affiliations:** Department of Applied Science, 63285 National Taitung University, 369, Section 2, University Road, Taitung 950309, Taiwan

## Abstract

A facile sensing assay was demonstrated for the selective and sensitive detection of thiram, using malic acid–functionalized silver nanoparticles (MA-AgNPs) as colorimetric probes. The factors affecting the synthesis of the MA-AgNPs were optimized and characterized by ultraviolet–visible spectroscopy, Fourier-transform infrared spectroscopy, transmission electron microscopy, and X-ray photoelectron spectroscopy. The MA-AgNPs were spherical, with an average diameter of 7.8 nm and a maximum absorbance at 390 nm. Further, the stability of the storage time, buffer pH, and salinity of the MA-AgNPs was investigated. The MA-AgNPs were employed to detect thiram in environmental samples. Good linearity of the calibration curve was achieved (0.1–1.0 ppm), with *R* ^2^ of 0.998 and a limit of detection (LOD) of 0.009 ppm. Additionally, the assay displayed good anti-interference toward thiram over other coexisting pesticides. More importantly, a smartphone-assisted colorimetric assay integrated with test paper was established for thiram detection, with an LOD of 0.71 ppm. This work shows the potential feasibility of the rational design for thiram detection in actual samples.

## Graphical abstract

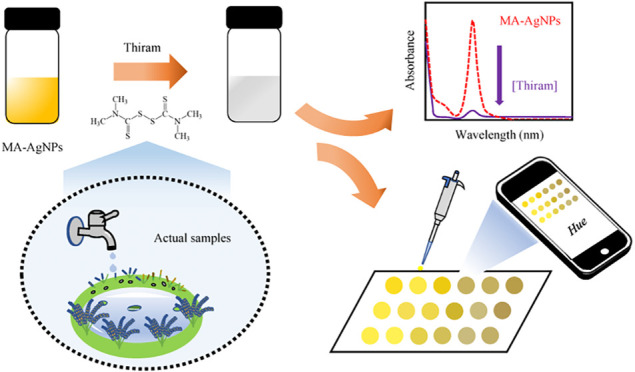

## 1. Introduction

Pesticides are crucial in modern agriculture for controlling and preventing the growth of weeds and pests.^1,2^ They help improve crop yield, quality, and shelf life. Among these pesticides, thiram (tetramethyl thiuram disulfide), as a dithiocarbamate and protective fungicide, has been extensively applied to protect crops from diseases and increase production.^3^ However, the high dose and irregular use of thiram have caused severe and unpredictable damage to the environment and human health, such as the death of chondrocytes, lymphocytes, and somatic cells.^4,5^ Resultantly, a facile and reliable analytical approach must be developed to accurately and quickly monitor thiram concentrations in food and environmental samples.

To date, various approaches have been established for the highly sensitive and selective detection of thiram, including high-performance liquid chromatography,^6^ liquid chromatography coupled to tandem mass spectrometry,^7,8^ surface-enhanced Raman scattering,^9,10^ and electrochemical assay.^10,11^ Although most of the above approaches demonstrate high sensitivity and selectivity, some limitations exist, such as the use of expensive equipment, time-consuming and tedious experimental processes, and skilled technicians. These drawbacks hinder their potential applications for rapid on-site testing.

Recently, metallic nanoparticle-based colorimetric assays have received considerable attention for identifying a wide variety of analytes in complex samples.^12–15^ This is owing to the advantages of simple preparation, ease of operation, low cost, high stability, good selectivity, and adequate sensitivity for rapid and visual analysis. Among these nanoparticles, silver nanoparticles (AgNPs) and gold nanoparticles (AuNPs) have gained significant attention owing to their distinctive plasmonic, electronic, and optical properties.^16,17^ In particular, they exhibit high molar extinction coefficients in the visible region and can be used as colorimetric probes.^18–22^ AgNPs possess characteristic localized surface plasmon resonance (LSPR) bands in the visible spectrum.^23^ The absorbance and wavelength can be adjusted by the size, shape, dielectric circumstance, and distance between the colloidal NPs. LSPR is a collective oscillation of the free electrons on the metal surface of NPs induced by the electromagnetic interaction of the metal with incident light of a specific wavelength. The LSPR absorption band of the yellow-colored AgNPs is located at approximately 390–410 nm in the visible spectrum. The color change of the NPs induced by the decrease in this characteristic absorption band and the shift to relatively high wavelengths is dependent on the analyte concentrations. Recently, several groups have successfully demonstrated AgNP-based colorimetric sensors for detecting pesticides in environmental samples.^24–27^ These research results have strongly proven the use of colorimetric strategies for pesticide detection with AgNPs as sensing probes; the strategies showed high selectivity and sensitivity and evident color changes were observed with the naked eye.

This study demonstrates a new colorimetric and visual sensing system for detecting thiram using malic acid (MA)–functionalized AgNPs (MA-AgNPs). The sensing mechanism is based on the aggregation of the MA-AgNPs due to the strong interactions between the S atoms on the thiram and the Ag atoms on the surface of the AgNPs. The color of the MA-AgNPs changes from light yellow to colorless. Thus, the colorimetric sensing assay using the MA-AgNPs is highly selective and sensitive. Additionally, a paper-based colorimetric approach is constructed, demonstrating that the images can be caught with a smartphone. Further, the image parameters can be converted into data information using ImageJ software for the visual and accurate detection of thiram. This shows the application potential of our colorimetric assay in the field of environmental safety.

## 2. Experimental Section

### 2.1. Chemicals and Materials

Silver nitrate (AgNO_3_) was purchased from Acros Organics (Geel, Belgium). MA was obtained from Alfa Aesar (Ward Hill, MA). Sodium borohydride (NaBH_4_), sodium chloride (NaCl), and pesticide standards were purchased from Sigma–Aldrich (St. Louis, MO). Tris­(hydroxymethyl)­aminomethane (Tris) was acquired from J. T. Baker (Phillipsburg, NJ). Hydrochloric acid (HCl) was obtained from Scharlau (Barcelona, Spain). All reagents and chemicals used in the current experimental setup were of analytical grade and required no further purification. Deionized water was used for solution preparation. Tris–HCl buffers were prepared at different pH levels according to our previously reported method.^26^

### 2.2. Instrumentations

The spectrum profile of AgNPs was characterized using an Analytikjena Specord 210 Plus (Analytik Jena, Jena, Germany). Capping-agent functional groups that stabilized the AgNP were investigated using a Fourier transform infrared (FTIR) spectrophotometer in the range of 4000–600 cm^–1^ (Frontier, PerkinElmer). The morphology and size of the AgNPs were investigated using a JEM-3010 transmission electron microscope (JEOL, Japan). Zeta potential was measured using a Zetasizer Nano ZS90 particle size analyzer (Malvern, UK). Surface composition and electronic structure analyses were performed by X-ray photoelectron spectroscopy (XPS) using a K-Alpha X-ray photoelectron spectrometer (Thermo Fisher Scientific, Waltham, MA) equipped with a monochromatic Al Kα X-ray source (1486.68 eV).

### 2.3. Preparation of Malic Acid–Functionalized Silver Nanoparticles

The MA-AgNPs were prepared using our previously reported protocol^28^ with slight modifications. Aqueous solutions of 2.0 mL of MA (2.5–15.0 mM), 6.0 mL of NaBH_4_ (2.5–20.0 mM), and 2.0 mL of AgNO_3_ (0.5–10.0 mM) were added to 20 mL glass vials with vigorous stirring for 1 h. The color of the solution mixture abruptly transformed from colorless to light yellow, indicating the formation of the MA-AgNPs. The synthesized MA-AgNPs were kept at room temperature for further use.

### 2.4. Colorimetric Detection of Thiram

The MA-AgNPs were processed to develop a colorimetric sensor for detecting thiram in an aqueous solution. Various concentrations of thiram (0.1–1.0 ppm) were mixed with the MA-AgNPs in the Tris–HCl buffer at pH 7.0. The color of the MA-AgNP solution changed from light yellow to colorless as the concentration of thiram was increased. A decreasing trend in the ultraviolet–visible (UV–Vis) spectra of the absorbance of the MA-AgNPs at 390 nm was observed after adding various concentrations of thiram. The calibration curve was recorded in the mentioned range with the respective changes in color. A linear plot was constructed by plotting the absorbance at 390 nm against the thiram concentrations. All experiments were repeated in triplicate. The limit of detection (LOD) was calculated using the following equation^29^

LOD=3σ/s

where σ is the standard deviation of the blank solution and s is the slope of the calibration curve.

### 2.5. Determination of Thiram in Actual Samples

Samples of rice, tap water, and lake water were used to investigate the analytical performance of the proposed assay. The rice samples were supplied by local farmers. Tap water and lake water samples were collected from our laboratory and Jinsin Lake at the National Taitung University (Taitung, Taiwan), respectively. The known concentration (50 μL, 100 ppm) of thiram was spiked in 10.0 mL of tap and lake water samples. The supernatant was collected by centrifugation at 4000 rpm for 5 min and filtered through a 0.22 μm nitrocellulose membrane to remove any suspended particles. First, the rice samples were crushed into homogenates. Thereafter, they (0.5 g) were added into a 15 mL centrifuge tube and spiked with a thiram solution (200 μL, 100 ppm). The solution was kept in a fume hood for 30 min, and 10 mL of deionized water was sequentially added. The mixture was vigorously shaken and extracted for 5 min; afterward, it was centrifuged at 12,000 rpm for 5 min. The supernatant extract was filtered using a 0.22 μm nitrocellulose membrane to remove any suspended particles. All the samples contained a final spiked concentration of 0.5 ppm thiram. The experimental results were measured in triplicate by UV–Vis spectroscopy. The concentration of thiram was quantified using a linear regression equation, and the percentage of recovery was calculated using the following equation^30^

recovery(%)=(calculated
thiram concentration/added thiram
concentration)×100

## 3. Results and Discussion

### 3.1. Synthesis of Malic Acid–Functionalized Silver Nanoparticles

Previous research results have revealed that the carboxyl and hydroxyl groups in the capping agents significantly influence the formation of AgNPs because of their chelation capability to silver ions.^31,32^ The AgNPs capped with specific molecules containing multiple carboxyl groups exhibited significant sensing activity, presumably because the AgNPs stabilized with specific molecules have considerably interacted with target analytes.^33^ MA (Figure S1) has two carboxyl groups with a relatively low molecular weight; thus, it was selected as the capping agent. The MA-AgNPs were synthesized through the chemical reduction of silver ions using NaBH_4_ with MA. The schematic representation of the MA-AgNP formation is illustrated in Scheme 1. The negatively charged hydrophilic carboxyl groups of MA on the surface of the AgNPs exhibited electric repulsion between the AgNPs and inhibited the aggregation of the AgNPs. Investigating the optimal conditions for synthesizing AgNPs is very important since the NPs require particular reaction conditions to form. Accordingly, the influences of experimental parameters (the concentrations of MA, AgNO_3_, and NaBH_4_) were examined systematically, and the results are illustrated in Figure S2. The optimal conditions were obtained by evaluating the absorbance and full width at half-maximum (fwhm) of the MA-AgNPs was calculated from their UV–Vis absorption spectra. According to the experimental results, 10.0 mM MA, 5.0 mM AgNO_3_, and 10.0 mM NaBH_4_ were the optimal conditions for the synthesis of the MA-AgNPs with high absorbance at 390 nm and low fwhm.

**Scheme 1:**
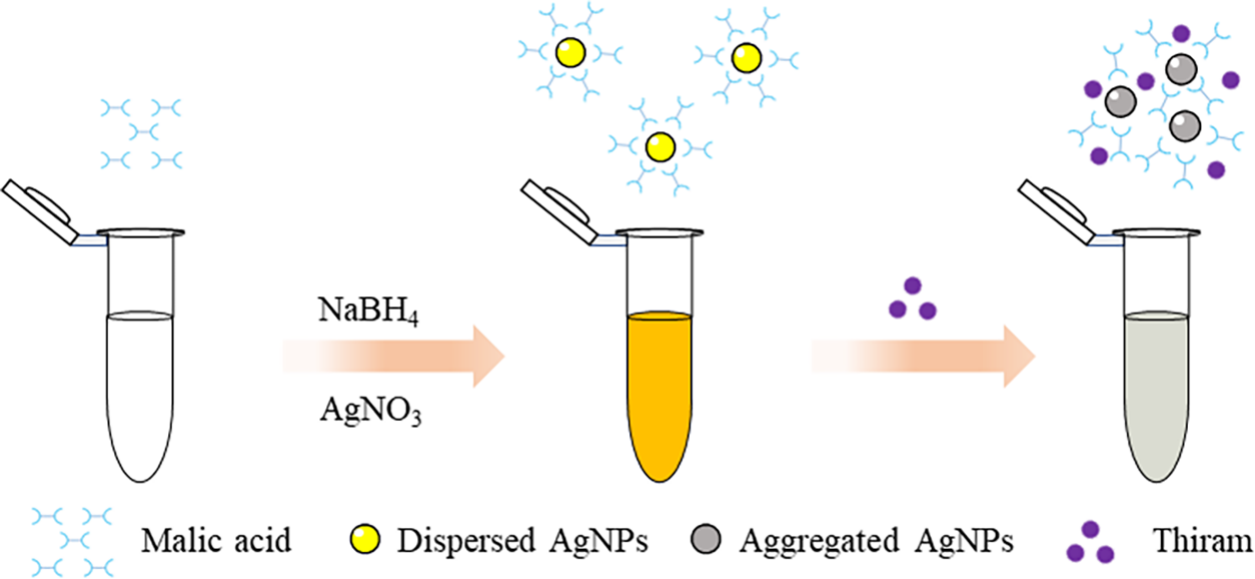
Schematic Illustration of the Colorimetric Determination of Thiram Based on the Aggregation of the Malic Acid–Functionalized Silver Nanoparticles

### 3.2. Characterization of Malic Acid–Functionalized Silver Nanoparticles

The MA-AgNPs were characterized using UV–Vis spectroscopy, FTIR spectroscopy, transmission electron microscopy (TEM), and XPS. The MA-AgNP solutions exhibited a vivid yellow color because of the LSPR, and a characteristic absorption peak at 390 nm was observed (Figure 1a). The results indicated the formation of stable and well-dispersed AgNPs; this was attributed to the electronic repulsion between the malate ions on the surface of the AgNPs. With the addition of thiram to the MA-AgNPs, the absorbance at 390 nm decreased dramatically, and the mixture turned colorless (Figure 1a). The observed phenomenon occurred because of the aggregation of the MA-AgNPs in the presence of thiram. Therefore, the size and morphology of the MA-AgNPs without and with thiram were verified by TEM. It was observed that the MA-AgNPs were well-dispersed and spherical, with an average diameter of 7.8 nm (Figure 1b). However, the aggregation of the MA-AgNPs was observed with 1.0 ppm of thiram (Figure S3). The X-ray diffraction patterns of MA and MA-AgNPs are shown in Figure S4. It shows the diffraction peaks at 2θ values of 38.2, 44.3, 64.4, and 77.6° were indexed to the (111), (200), (220), and (311) lattice planes of the face-centered cubic (FCC) structure of AgNPs, respectively.^34–36^

**Figure 1:**
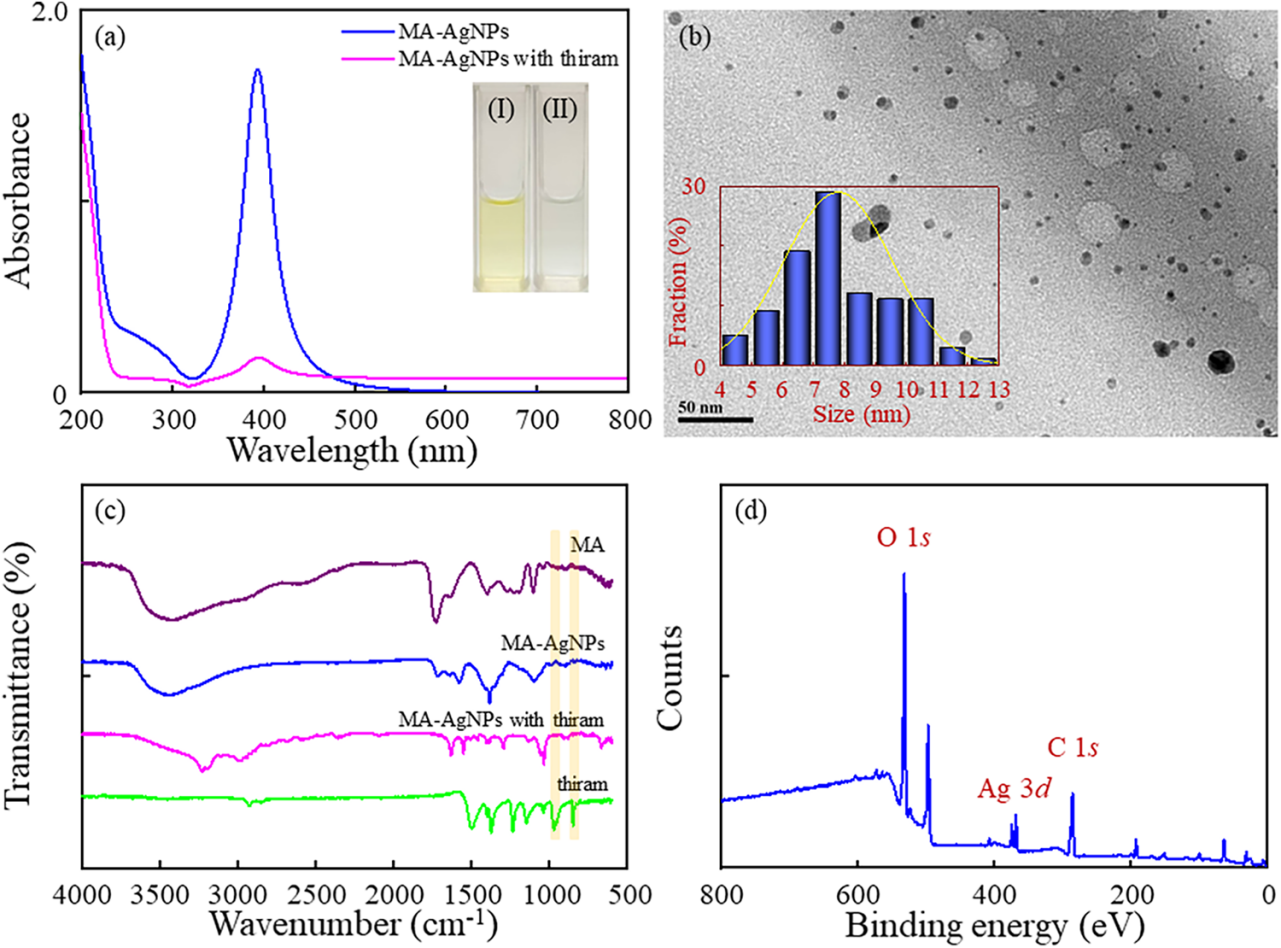
(a) Ultraviolet–visible (UV–Vis) absorption spectra of the malic acid–functionalized silver nanoparticles (MA-AgNPs) and the MA-AgNPs with 1.0 ppm thiram. Inset: corresponding photographs of the (I) MA-AgNPs and (II) MA-AgNPs with thiram. (b) The transmission electron microscopy image of the MA-AgNPs. Inset: the particle size histogram. (c) Fourier transform infrared spectra of the MA, MA-AgNPs, MA-AgNPs with thiram, and thiram. (d) X-ray photoelectron spectroscopy survey spectrum of the MA-AgNPs.

The surface modification of the AgNPs with MA was further confirmed by FTIR spectroscopy. Figure 1c shows the IR spectra of the MA and MA-AgNPs. The assignment of FTIR peaks is summarized in Table S1. The absorption bands of the functional groups of pure MA can be assigned as follows. The broad band at 3600–2700 cm^–1^ represented the stretching vibration of the hydroxyl group.^37^ The two characteristic bands at 1630–1399 cm^–1^ belonged to the antisymmetric and symmetric stretching vibrations of the carboxylate group.^38^ The peak at 1729 cm^–1^, representing the stretching vibration of the C = O in the carboxyl group^39^ became weaker than that of MA after capping on the AgNPs, and it slightly shifted to a lower wavenumber. These peaks appeared owing to the formation of the MA-AgNPs.

XPS was used to characterize the surface composition, bonding environment, and electronic states of the MA-AgNPs. In the survey spectrum of the MA-AgNPs, three major peaks were observed at 285.1, 368.1, and 532.1 eV for C 1s, Ag 3d, and O 1s with atomic percentages of 17.83%, 11.48%, and 67.96%, respectively (Figure 1d). The survey spectrum was deconvoluted and analyzed to identify the chemical nature of the elements. The matching peaks of C–C, C–O, and O–C = O are shown in Figure S5a with binding energies of 284.8, 286.4, and 288.4 eV, respectively.^40^ The Ag 3d XPS spectrum (Figure S5b) revealed two characteristic peaks at 368.0 and 374.0 eV, corresponding to a splitting doublet (i.e., 3d_5/2_ and 3d_3/2_, respectively). The splitting doublet between the 3d_5/2_ and 3d_3/2_ peaks was exactly 6.0 eV, indicating the presence of metallic silver (Ag^0^) on the surface.^41^ The fitted O 1*s* spectrum was consistent with the C–O at 531.8 eV, and a small subpeak at 535.8 eV was characterized as the adsorbed water (Figure S5c).^42^ These spectra support the presence of MA and Ag on the nanocomposite surface.

### 3.3. Stability of the Malic Acid–Functionalized Silver Nanoparticles

The stability of the MA-AgNPs was evaluated to determine their potential applicability. The MA-AgNPs showed excellent stability when stored at 4 °C for 60 days, revealing no aggregation of NPs, as reflected from the SPR band having no change in absorbance and wavelength (Figure S6a). The absorbance at 390 nm was slightly decreased, which may be because of the diffusion of the stabilizing molecules from the surfaces of the AgNPs and the subsequent changes in their shape and size.^43^ Similarly, the SPR bands of the MA-AgNPs were monitored at various pH (5.0–9.0). As shown in Figure S6b, the MA-AgNPs remain stable at pH 7.0–9.0; however, a decrease in the SPR bands is observed in acidic pH (5.0 and 6.0). This may be because the surface charges (which keep the NPs well-dispersed in solutions and regulate electrostatic repulsion between each NP) of the NPs were altered in highly acidic and basic media, resulting in little aggregation of the NPs. Additionally, the stability of the MA-AgNPs was evaluated in various NaCl concentrations (0–20.0 mM). The MA-AgNPs were stable at 0–20 mM NaCl because the absorption spectra were almost unchanged (Figure S6c). Further, excellent batch-to-batch reproducibility was obtained (Figure S6d).

### 3.4. Detection Mechanism for Sensing Thiram

According to previous studies, colorimetric assays for pesticide detection using AgNPs as probes showed that the surface modification of the AgNPs remarkably affected the detection performance.^27,33^ In this study, the absorbance at 390 nm decreased dramatically, and the color of the solution changed from light yellow to colorless (Figure 1a, inset) with the addition of thiram. The sulfur atoms on the thiram molecules have a high affinity for the surface of AgNPs because of the electrostatic attraction from lone pair electrons on the sulfur atom to the silver atom.^27^ Thus, the sensing mechanism could be explained as the aggregation of the AgNPs induced by thiram (Figure S7). According to the simulation results of the previous literature,^44,45^ thiram tends to interact with AgNPs via central sulfur atoms through electron donation (AgNPs as electrophile). Additionally, Ag is considered a soft acid, and thiram can be regarded as a soft molecule; therefore, soft–soft interactions are expected to play a significant role in these systems.^44^ The FTIR spectra provided evidence for these interactions. The intense peaks at 848 cm^–1^ and 970 cm^–1^ corresponding with the stretching vibrations of CH_3_N and C–S, respectively, disappeared, as shown in Figure 1c, and the result indicated the interactions between the C–S bonds and the Ag surface. Similar results were obtained in the previous studies.^46,47^

Furthermore, the aggregation of the MA-AgNPs was identified by TEM and zeta potential measurements. The TEM images of the MA-AgNPs after adding thiram showed that the MA-AgNPs aggregated, compared with the initial well-dispersed state (Figure S3). The measured zeta potential of the MA-AgNPs (Figure S8) was changed from −37.2 to −22.9 mV after the addition of thiram, demonstrating a decrease in the negative charges on the surface of the MA-AgNPs. The reduced electrostatic repulsion led to the aggregation of the MA-AgNPs. The TEM and zeta potential measurement results confirmed the aggregation of the MA-AgNPs with thiram.

### 3.5. Evaluation of the Selectivity, Interference, Sensitivity, and Stability of the Assay

The synthesized MA-AgNPs served as a highly selective colorimetric probe for detecting thiram. Considering the potential interference from other pesticides in environmental samples, a series of tests were performed to evaluate the selectivity of the proposed method. The MA-AgNPs were mixed with various pesticides at a concentration of 1.0 ppm. They included trichlorfon, dichlorvos, chlorothalonil, chlorpyrifos, clothianidin, glufosinate-ammonium, propanil, glyphosate, dicofol, fenvalerate, thiodicarb, acetamiprid, kresoxim-methyl, pencycuron, profenofos, imidacloprid, bifenthrin, and thiram. Only thiram induced a significant color change of the MA-AgNP solution, from light yellow to colorless (Figure 2). The absorption spectra were monitored to further confirm the assay’s selectivity (Figure S9a). The characteristic absorption peak at 390 nm revealed a dramatic decrease in its absorbance with the addition of thiram. The initial light yellow color indicated that the MA-AgNPs were in their dispersed state. With the interaction of thiram with the MA-AgNPs, the NPs aggregated. The decrease in the absorbance and the color change were due to the aggregated state of the NPs.

**Figure 2:**
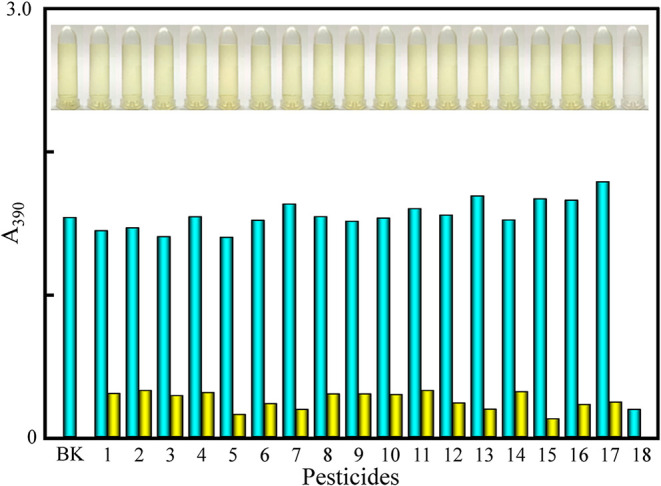
Absorbance at 390 nm of the MA-AgNPs for thiram over other pesticides (blue bar) and the mixture of thiram with other pesticides (yellow bar). BK: blank. 1–18 are various pesticides (i.e., trichlorfon, dichlorvos, chlorothalonil, chlorpyrifos, clothianidin, glufosinate-ammonium, propanil, glyphosate, dicofol, fenvalerate, thiodicarb, acetamiprid, kresoxim-methyl, pencycuron, profenofos, imidacloprid, bifenthrin, and thiram). Inset: Images representing the color variations of the MA-AgNPs induced by the addition of different pesticides.

Further, an interference study was conducted to determine the specificity of the assay for determining thiram using the MA-AgNPs. For this, various pesticides were tested to ensure that they might not interfere with the thiram detection under the optimized conditions. The absorbance of the tested pesticides with the MA-AgNPs showed similar responses as those of the MA-AgNPs and thiram, demonstrating that the presence of these pesticides did not interfere in the detection of thiram (Figures 2 and S9b).

The reaction buffer pH and incubation time were investigated to achieve high sensing performance for the thiram-detection assay. The absorbance of the MA-AgNPs at 390 nm was monitored to select the optimal conditions. According to the experimental results shown in Figure S10, a buffer pH of 7.0, 25 °C, and an incubation time of 60 min were selected for further experiments. Under the optimized conditions, the thiram sensing performance was evaluated using the MA-AgNPs as colorimetric probes. After incubation with the premixed solution and various concentrations of thiram, the UV–Vis absorption spectra of the MA-AgNPs were recorded. The absorbance at 390 nm was observed to significantly decrease for the MA-AgNPs with the increase in thiram concentrations (Figure 3). The linear regression equation was *A* = −1.62693 [thiram (ppm)] + 1.7833, with the coefficient of determination (*R* ^2^) = 0.998, where *A* represents the absorbance at 390 nm of the MA-AgNPs incubated with thiram concentrations of 0.1–1.0 ppm. Furthermore, the calculated LOD was 0.009 ppm (3σ/s). The Taiwan Food and Drug Administration has prescribed that the maximum residue limit (MRL) of thiram in rice samples is 0.79 ppm.^48^ U.S. Environmental Protection Agency has prescribed a limit of 7 ppm in fruits and European Union has established the MRL of 5 ppm for thiram in apples and pears.^49^ Table 1 summarizes the comparison of the LODs of the proposed colorimetric assay for thiram detection with those of previous studies. The proposed assay demonstrated detection range and LOD comparable to those of previous studies for detecting thiram with high selectivity.

**Figure 3:**
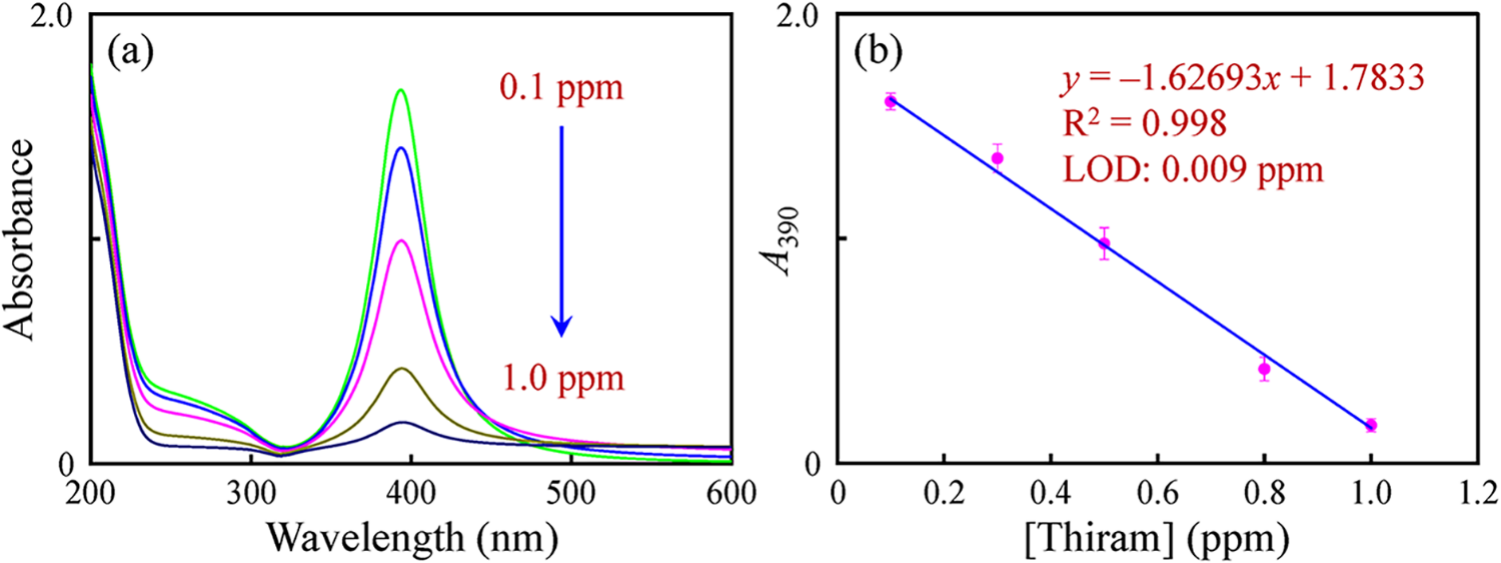
(a) UV–Vis absorption spectra of the MA-AgNPs with different concentrations (0.1–1.0 ppm) of thiram; (b) the plot of the absorbance at 390 nm against the concentration of thiram and its linear calibration plot.

**Table 1:** Comparison of the Proposed Method with Previously Reported Colorimetric Methods for Detecting Thiram^a^

probes	linear range (ppm)	LOD (ppb)	selectivity	applications	refs
CTAB-AuNPs	0.048–2.404	41		tomato, cucumber, watermelon, river water, tap water	^50^
Au nanobipyramids	0.005–0.289	4	10 pesticides, 9 metal ions	apple, black tea	^51^
PHMB-AgNPs	0.024–24.044	9	5 pesticides	lake water	^27^
AuNPs@4-ABT/Ag^+^	0.01–0.48	10	8 pesticides, 3 metal ions, BSA, glucose	apple, soil	^52^
Ag triangular nanoplates	0.048–0.120	31	7 pesticides	wheat	^53^
Si-AgNPs	2.38–41.84	51	7 pesticides, 5 metal ions, 5 anions	river water, tap water, apple, guava, broad beans, green beans, soil	^54^
MA-AgNPs	0.1–1.0	9	18 pesticides	rice, tap water, lake water	this work

a 4-ABT: 4-aminothiophenol; BSA: bovine serum albumin; CTAB: cetyltrimethylammonium bromide; MA: malic acid; PHMB: polyhexamethylene biguanide hydrochloride; Si: 3-glycidoxypropyltrimethoxysilane.

The stability of the proposed method was checked by performing five replicate measurements for 1.0 ppm thiram over a single day (intraday analysis, *n* = 5) and after 5 days (interday analysis, *n* = 5). The obtained results (Figure S11) showed an excellent reproducibility, with RSDs of 2.7 and 5.9% for interday and intraday analyses, respectively. Therefore, the developed nanosensor is reproducible and accurate.

### 3.6. Smartphone-Integrated Test Papers for Detecting Thiram

In practical applications, a class of portable diagnostic devices, such as paper-based colorimetric nanosensors, has shown significant potential for pesticide detection.^55,56^ Based on its visual changes in color, low-cost, and portable features, a paper-based colorimetric sensing device was developed for thiram detection. The assembly process of the test paper is illustrated in Scheme 2. First, different concentrations (10 μL, 1.0–10.0 ppm) of thiram were dropped on circular absorbent papers (6 mm diameter) and dried in an oven at 60 °C for 10 min. Thereafter, the sensing probes (20 μL MA-AgNPs) were dropped to react with thiram for 10 min, and the test papers were dried in an oven at 60 °C for 5 min. The yellow color of the test papers changed to dark yellow with the increase in the thiram concentrations. All of the different colors were captured using a smartphone, and the captured images were transferred using ImageJ to the split-spectrum Hue-saturation-brightness color space,^57^ thereby enabling the measurement of the mean Hue values. A linear calibration curve (Figure 4) was constructed using the Hue values as a function of thiram concentrations ranging from 1.0 to 10.0 ppm, with the R^2^ of 0.991. The LOD for the paper-based assay for detecting thiram was calculated to be 0.71 ppm (3σ/s).

**Figure 4:**
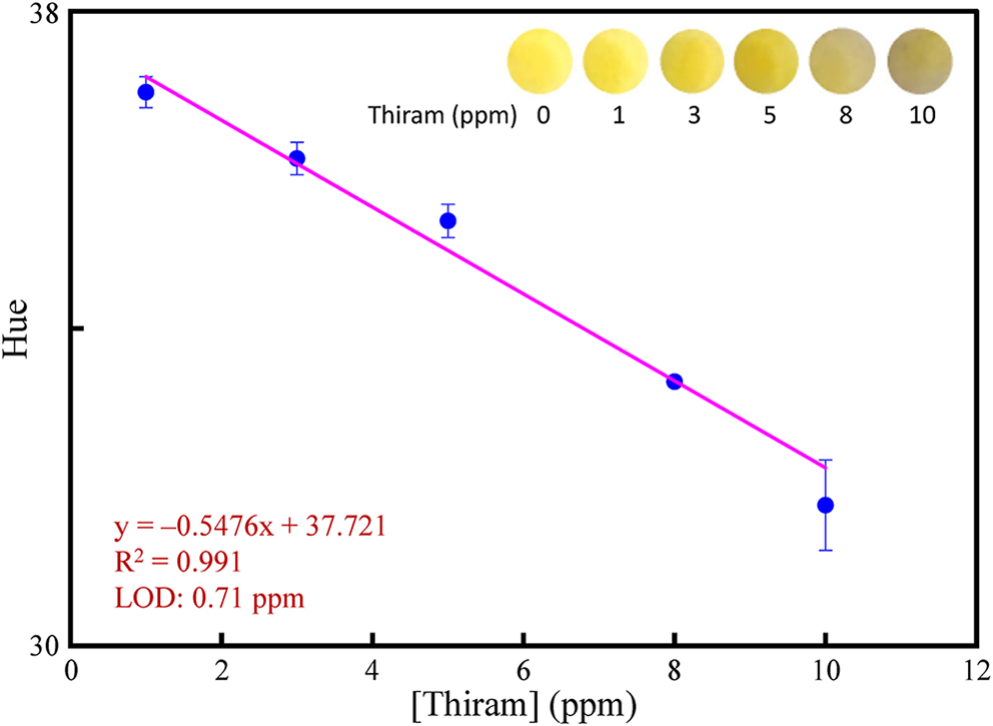
Linear responses of the color of the MA-AgNPs-based test paper to the concentrations of thiram. The inset shows the corresponding color of the test paper.

**Scheme 2:**
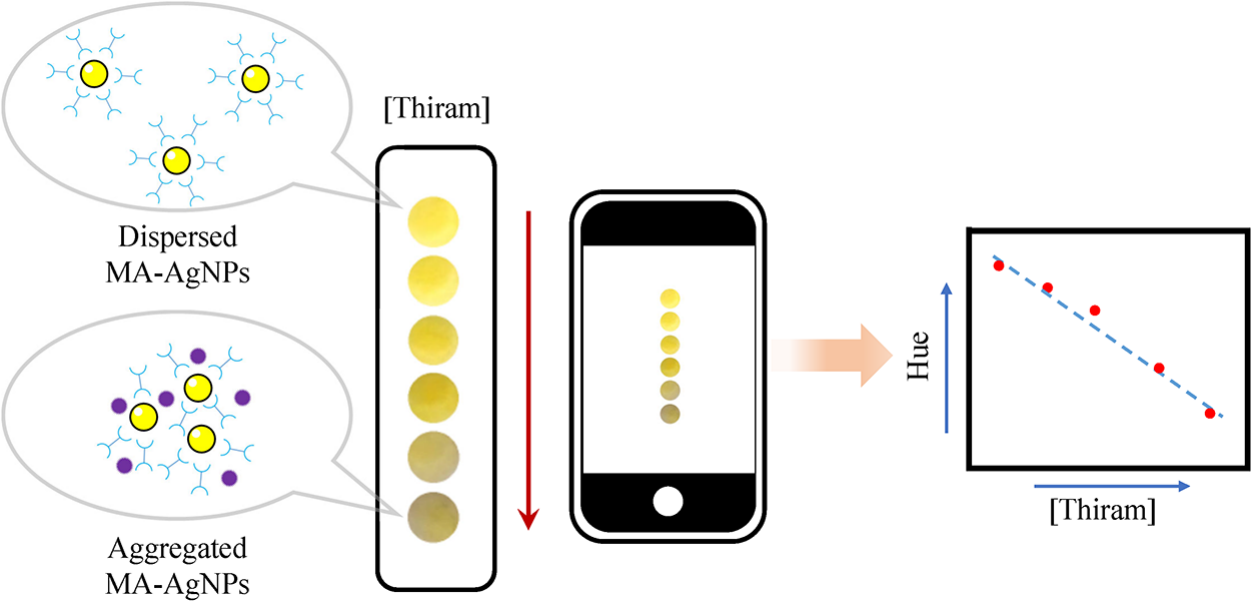
Smartphone-Assisted Colorimetric Sensing Platform Integrated with Test Papers for Detecting Thiram

### 3.7. Analysis of Actual Samples

The assay’s applicability to detecting thiram using the MA-AgNPs as colorimetric probes was investigated in tap water, lake water, and rice samples. Figure S12 illustrates the UV–Vis absorption spectra of the colorimetric assay for analyzing these samples using the MA-AgNPs. They were spiked with a known thiram concentration via the protocol described in Section 2.5 and analyzed with the MA-AgNPs. As shown in Table 2, the recovery of the assay is determined to be within the range of 94.6–98.8% with a relative standard deviation (RSD) of less than 8.9%. The satisfactory recoveries indicate that the MA-AgNPs exhibited high precision and accuracy in determining thiram. Therefore, the developed MA-AgNPs used as colorimetric probes are suitable for detecting thiram with high sensitivity, selectivity, and stability.

**Table 2:** Determination of Thiram in Actual Samples

sample	spiked (ppm)	measured (ppm)	recovery (%)	RSD (%)
rice 1	0.5	0.494	98.8	1.3
rice 2	0.5	0.480	96.0	5.0
rice 3	0.5	0.477	95.4	0.9
tap water	0.5	0.473	94.6	8.9
lake water	0.5	0.487	97.4	4.9

## 4. Conclusions

The present study demonstrated the use of the MA-AgNPs for an efficient colorimetric and paper-based assay for the specific detection of thiram. Multiple studies, such as UV–Vis spectroscopy, TEM, and zeta potential measurements, provided evidence to support that the addition of thiram induced the aggregation of the MA-AgNPs, combined with the color change from light yellow to colorless in the colloidal solution. The proposed colorimetric assay presented a direct and visually perceptible method for thiram detection, whereas the paper-based sensing approach offered practicality and ease through the integration of smartphones for on-site analysis. The assay demonstrated satisfactory sensitivity with low LODs of 0.009 and 0.71 ppm for colorimetric and paper-based sensing, respectively. The recovery of the assay was determined to be within the range of 94.6–98.8%, with an RSD of less than 8.9%, indicating the accuracy and precision of the proposed method. The proposed colorimetric assay for thiram detection exhibited good selectivity and sensitivity and was successfully applied to determining thiram in environmental samples. By combining the test papers with a smartphone, a visual and convenient colorimetric assay was proposed, which can be utilized in thiram detection in practical applications.

## Supplementary Material



## Notes

Chemical structure of malic acid; optimization of various experimental parameters on the preparation of MA-AgNPs; TEM images; XRD patterns; XPS spectra of C 1s, Ag 3d, and O 1s of the MA-AgNPs; effect of storage time, pH, and ionic strength on the MA-AgNPs; sensing mechanism; Zeta potential of the MA-AgNPs and MA-AgNPs with thiram; selectivity and inference of the MA-AgNPs; intraday and interday analyses; UV–Vis absorption spectra of the thiram-spiked actual samples; peak assignments for the FTIR spectra (PDF)
