# Supplementary material for: Highly Selective Colorimetric and Smartphone-Based Paper Assay Using Malic Acid–Functionalized Silver Nanoparticles for Thiram Detection
Source: ACS Omega. 2025 May 13;10(20):20435–43. doi: 10.1021/acsomega.5c00575 (PMC12120599; doi:10.1021/acsomega.5c00575)
Supplement: Supplementary file 1 [file ao5c00575_si_001.pdf]

Supporting Information (SI)

Highly Selective Colorimetric and Smartphone-Based Paper Assay Using Malic Acid-Functionalized Silver Nanoparticles for Thiram Detection

Kuan-Hsun Chen, Wei-Yu Wang, Cho-Chun Hu, and Tai-Chia Chiu*

Department of Applied Science, National Taitung University, Taitung 950309, Taiwan

Correspondence:
Professor Tai-Chia Chiu, Department of Applied Science, National Taitung University, 369, Section 2, University Road, Taitung 950309, Taiwan
Tel.: 886-89-517990; fax: 886-89-518108
E-mail: tcchiu@nttu.edu.tw

E-mail addresses: ken921131212@gmail.com (K.-H. Chen), xx38084013@gmail.com (W.-Y. Wang), cchu@nttu.edu.tw (C.-C. Hu)

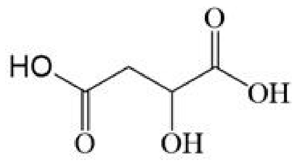

Figure S1. Chemical structure of malic acid.

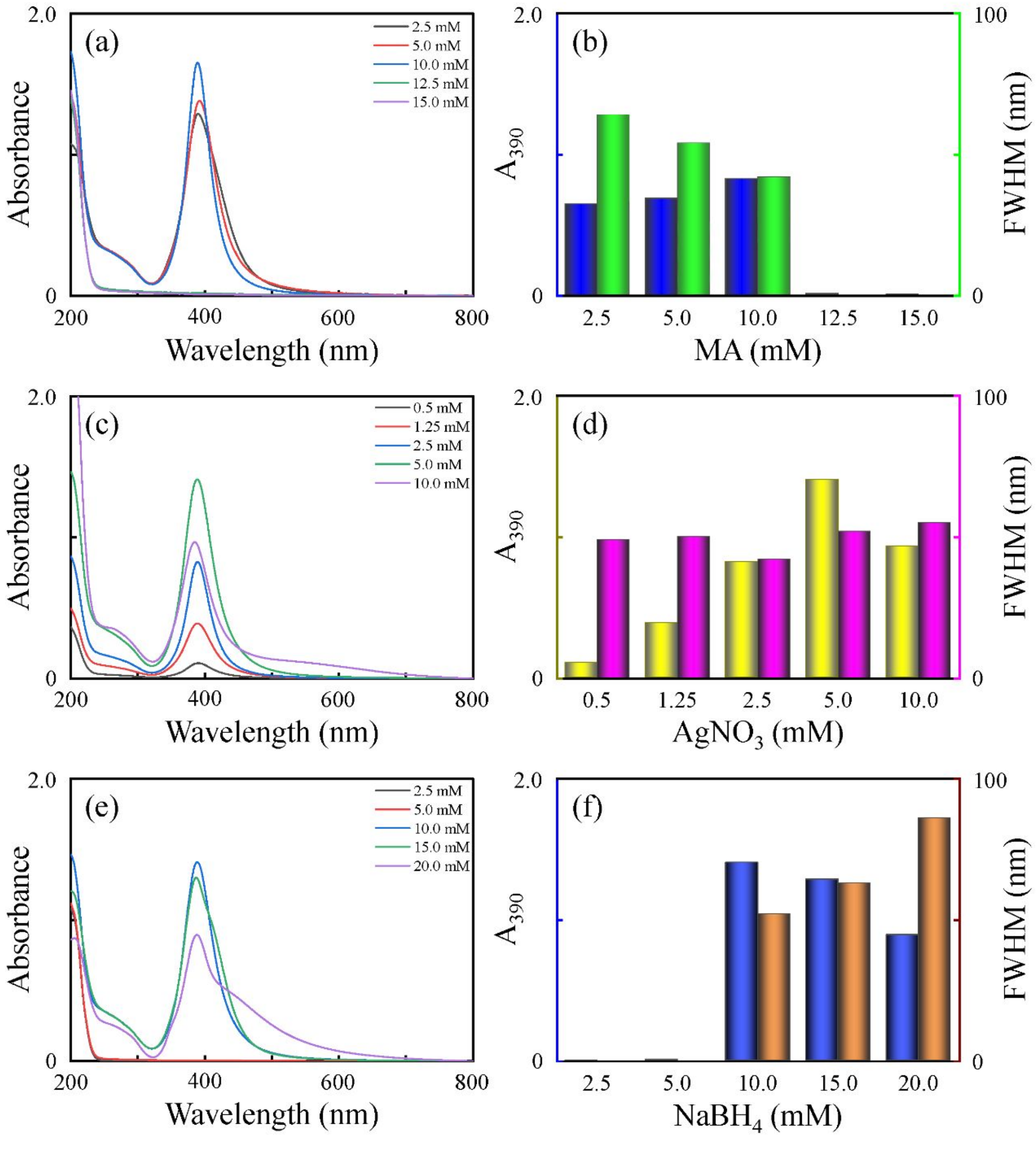

Figure S2. Ultraviolet‐visible (UV–Vis) absorption spectra and bar charts of various experimental parameters on the synthesis of the malic acid–functionalized silver nanoparticles (MA-AgNPs): concentrations of (a, b) MA, (c, d) $AgNO_3$, and (e, f) $NaBH_4$.

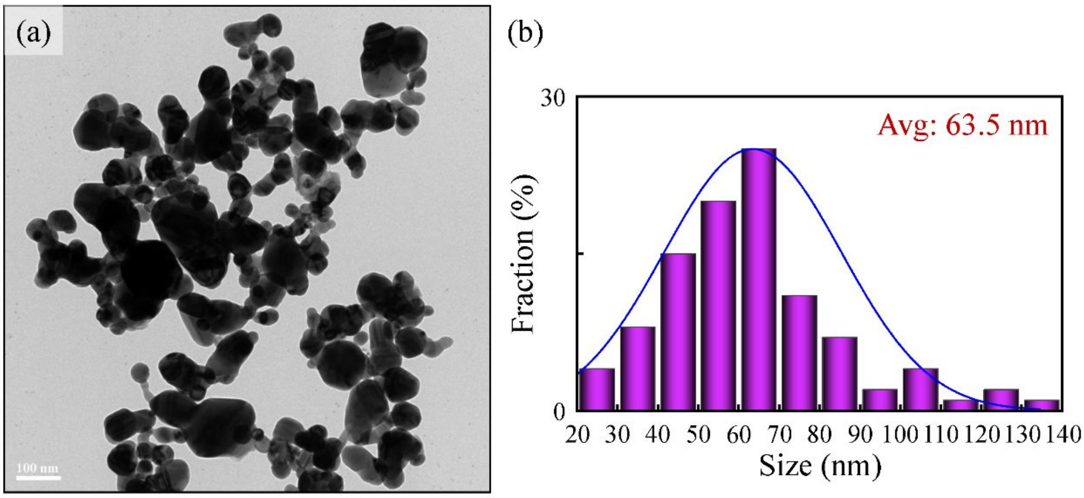

Figure S3. (a) The TEM image and (b) the particle size histogram of the MA-AgNPs with 1.0 ppm thiram.

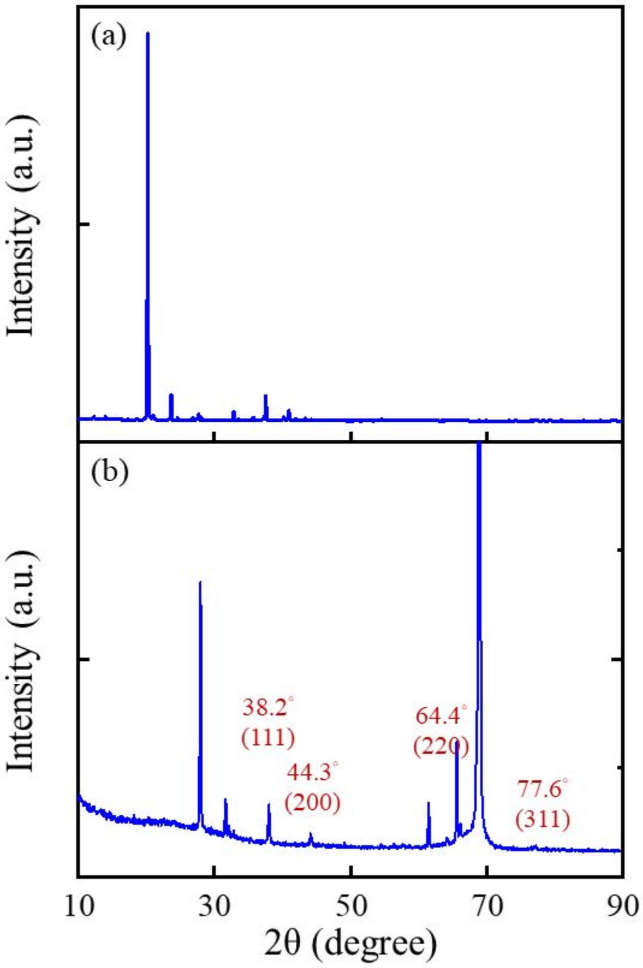

Figure S4. XRD patterns of (a) MA and (b) MA-AgNPs.

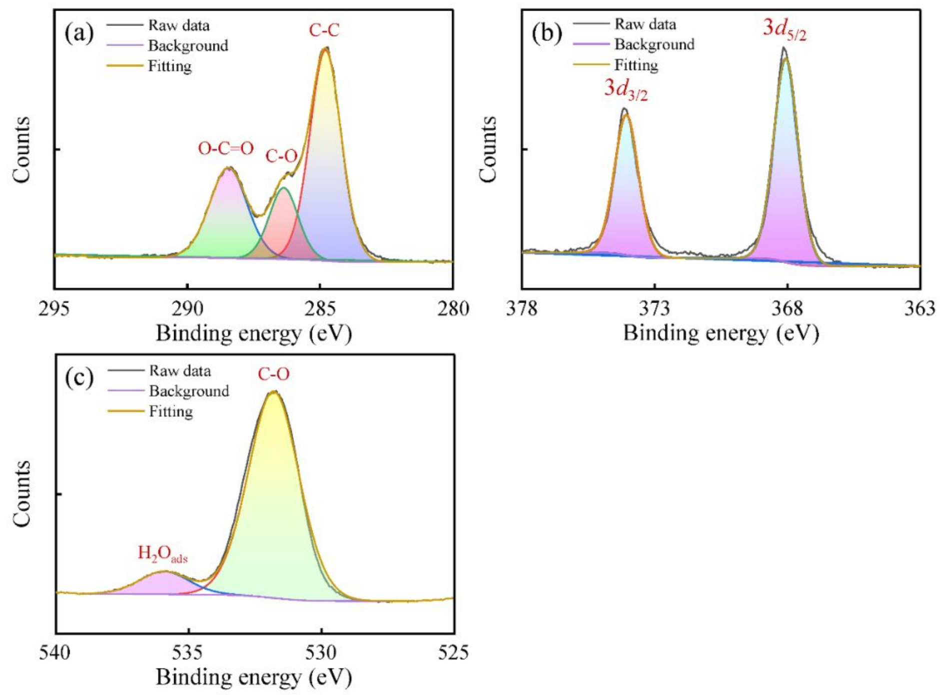

Figure S5. High-resolution (a) C 1*s*, (b) Ag 3*d*, and (c) O 1*s* X-ray photoelectron spectra of the MA-AgNPs.

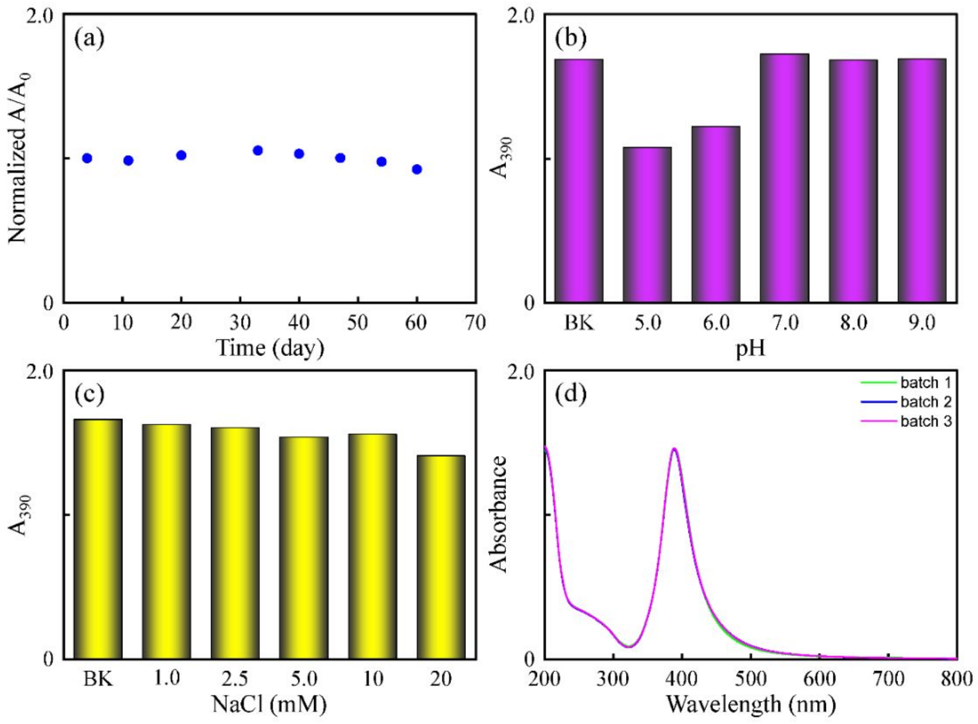

Figure S6. Effect of (a) storage time, (b) pH, and (c) ionic strength on the absorbance at 390 nm of the MA-AgNPs. (d) The batch-to-batch reproducibility for the synthesis of the MA-AgNPs under optimal conditions.

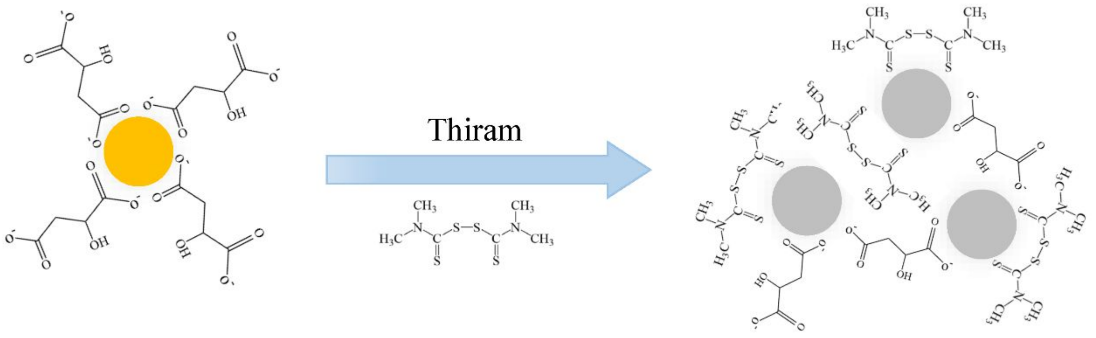

Figure S7. Sensing mechanism and interaction between thiram and MA-AgNPs.

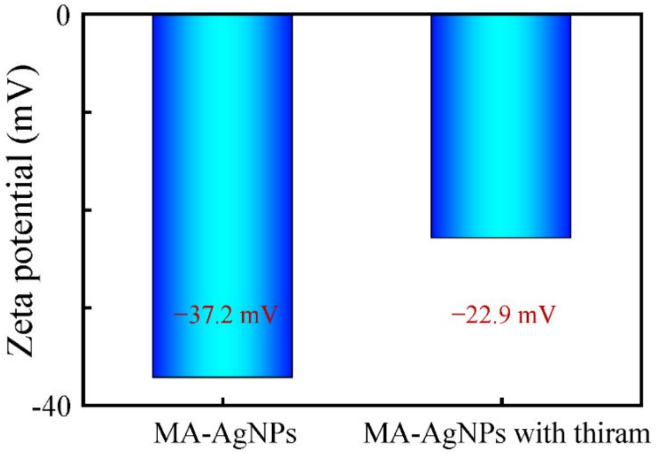

Figure S8. Zeta potential measurements of the MA-AgNPs and MA-AgNPs with 1.0 ppm thiram.

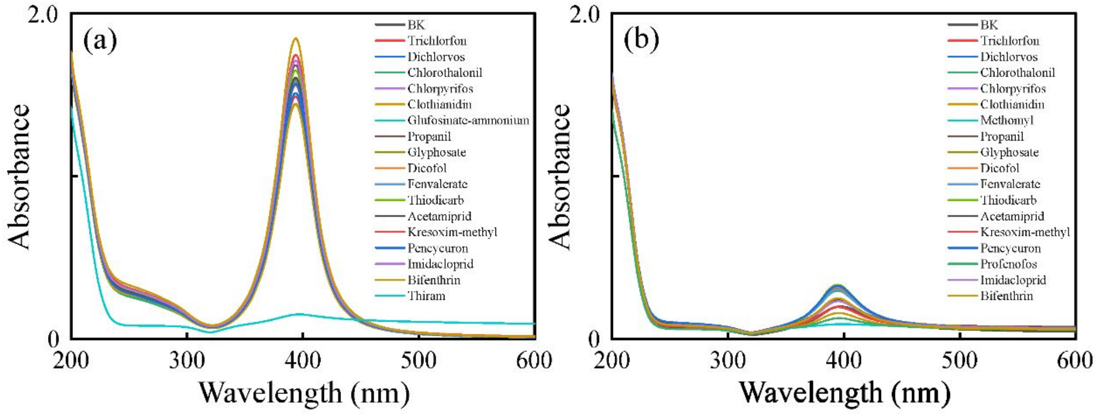

Figure S9. (a) Selectivity and (b) interference study results of the effect of various pesticides via UV–Vis absorption spectra.

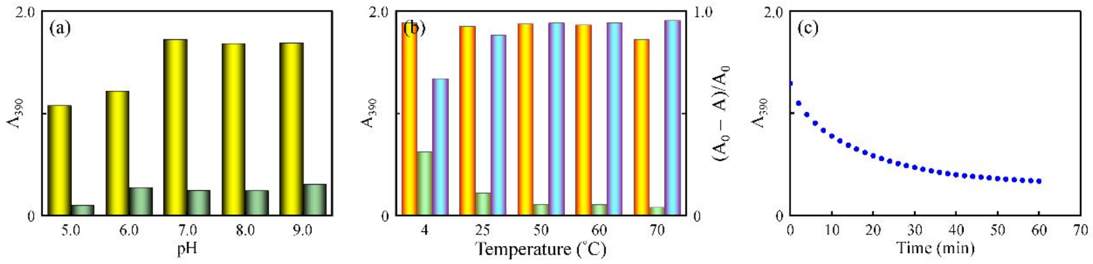

Figure S10. (a) Effect of pH on the absorbance at 390 nm of the MA-AgNPs without (yellow bars) and with (green bars) 1.0 ppm thiram. (b) Effect of temperature on the absorbance at 390 nm of the MA-AgNPs without (yellow bars) and with (green bars) 1.0 ppm thiram, and $(A_0 - A)/A_0$ (blue bars). (c) Effect of reaction time on the absorbance at 390 nm of the MA-AgNPs with 1.0 ppm thiram.

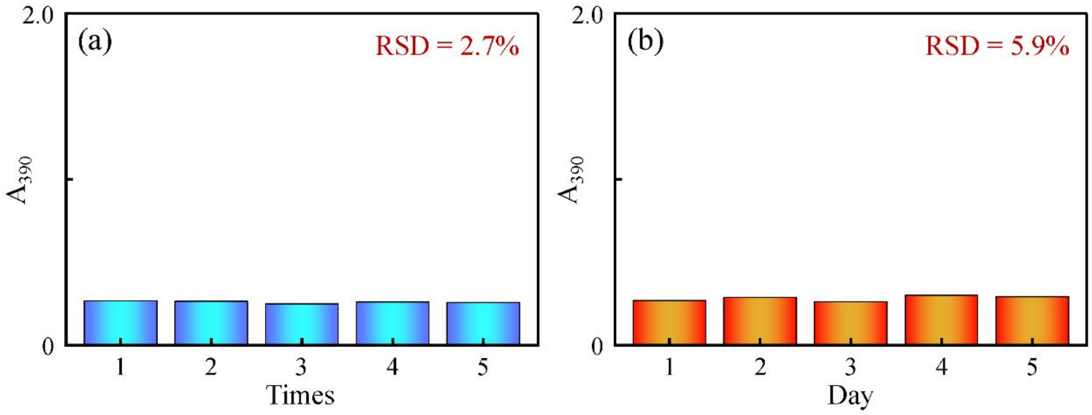

Figure S11. (a) Intraday and (b) interday analyses (n = 5) of the proposed method.

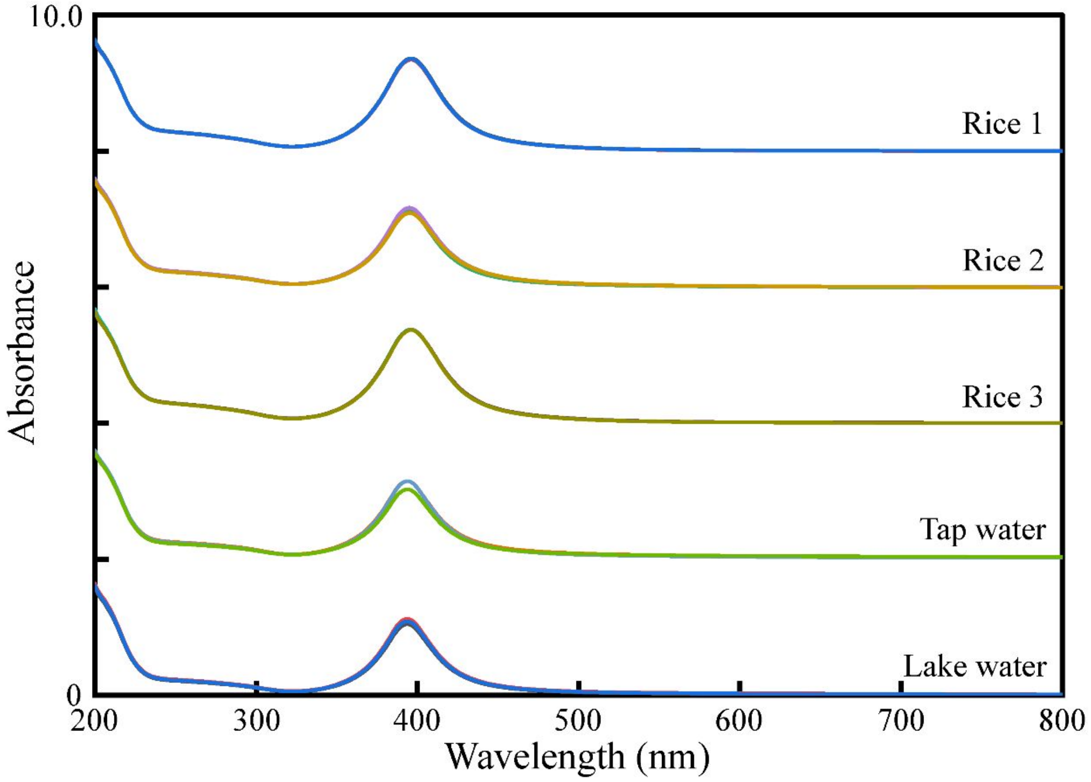

Figure S12. UV–Vis absorption spectra for the detection of spiked thiram in various actual samples (n = 3).

Table S1 Peak assignments for the FTIR spectra of MA, MA-AgNPs, MA-AgNPs with thiram and thiram.

	Wavenumber (cm $^{-1}$)	Peak assignment
MA	2700~3600	O–H stretching
	1630~1399	C=O antisymmetric and symmetric stretching
	1729~1716	C=O stretching
	1274	C–O stretching
	1220	C–H in-plane deformation
	1105~1098	C–OH
MA-AgNPs	1384	C–H symmetric deformation
	1576	bidentate nitrate
MA-AgNPs with thiram	2991	C–H stretching
	1295	CH_2 rocking
	1037	C=S stretching
	688	C–C–C in-plane bending
Thiram	2926	CH_2 asymmetrical stretching
	2865	CH_2 symmetrical stretching
	1500	C–H bending
	1374	C-N linked to methyl group
	1235	HCN
	1147	CNC
	970	C–S stretching
	848	CH_3N stretching
